# First records of *Secretargas transgariepinus* (Argasidae) in Libya and Jordan: corrections of collection records and detection of microorganisms

**DOI:** 10.1007/s00436-024-08239-5

**Published:** 2024-05-28

**Authors:** Martin Ševčík, Eva Špitalská, Michaela Maliterná, Peter Kabát, Petr Benda

**Affiliations:** 1 Institute of Virology, Biomedical Research Center, Slovak Academy of Sciences, Dúbravská cesta 9, SK–845 05 Bratislava, Slovakia; 2 Department of Microbiology and Virology, Faculty of Natural Sciences, Comenius University, Ilkovičova 6, SK–842 15 Bratislava, Slovakia; 3 Department of Zoology, National Museum (Natural History), Václavské nám. 68, CZ–115 79 Praha 1, Czech Republic; 4 Department of Zoology, Faculty of Science, Charles University, Viničná 7, CZ–128 43 Praha 2, Czech Republic

**Keywords:** Secretive bat-argas, Nidicolous, Arid area, Main host, Virus, Microorganism, Middle East

## Abstract

The primarily bat-associated argasid tick, *Secretargas transgariepinus* (White, 1846), is a member of the Afrotropical and southern Palaearctic fauna. Probably because of its secretive life style, little is known about this species and records of its collection are scant. Based on morphological revisions of the available specimens, we report new Middle Eastern records for this tick species that had been misidentified as other bat-associated argasid taxa. These specimens are larvae from three localities, and represent the first records of *S*. *transgariepinus* from two countries: one larva from Sabratha (Libya) was collected from an unidentified bat species (possibly *Eptesicus isabellinus*), seven larvae from Azraq-Shishan (Jordan), and 78 larvae from Shamwari (Jordan) were all collected from *Otonycteris hemprichii*. Twenty larvae from Shamwari were also tested for the presence of both, viral or bacterial microorganisms by PCR. Three ticks were found to be infected with the Murid gammaherpesvirus 68 (MHV-68), one with *Borrelia burgdorferi* sensu lato, and four with a *Rickettsia* sp. closely related to *Rickettsia slovaca*. The findings represent a first evidence for the occurrence of these possible pathogens in *S*. *transgariepinus.*

**Supplementary Information:**

The online version contains supplementary material available at 10.1007/s00436-024-08239-5.

## Introduction

**Fig. 1:**
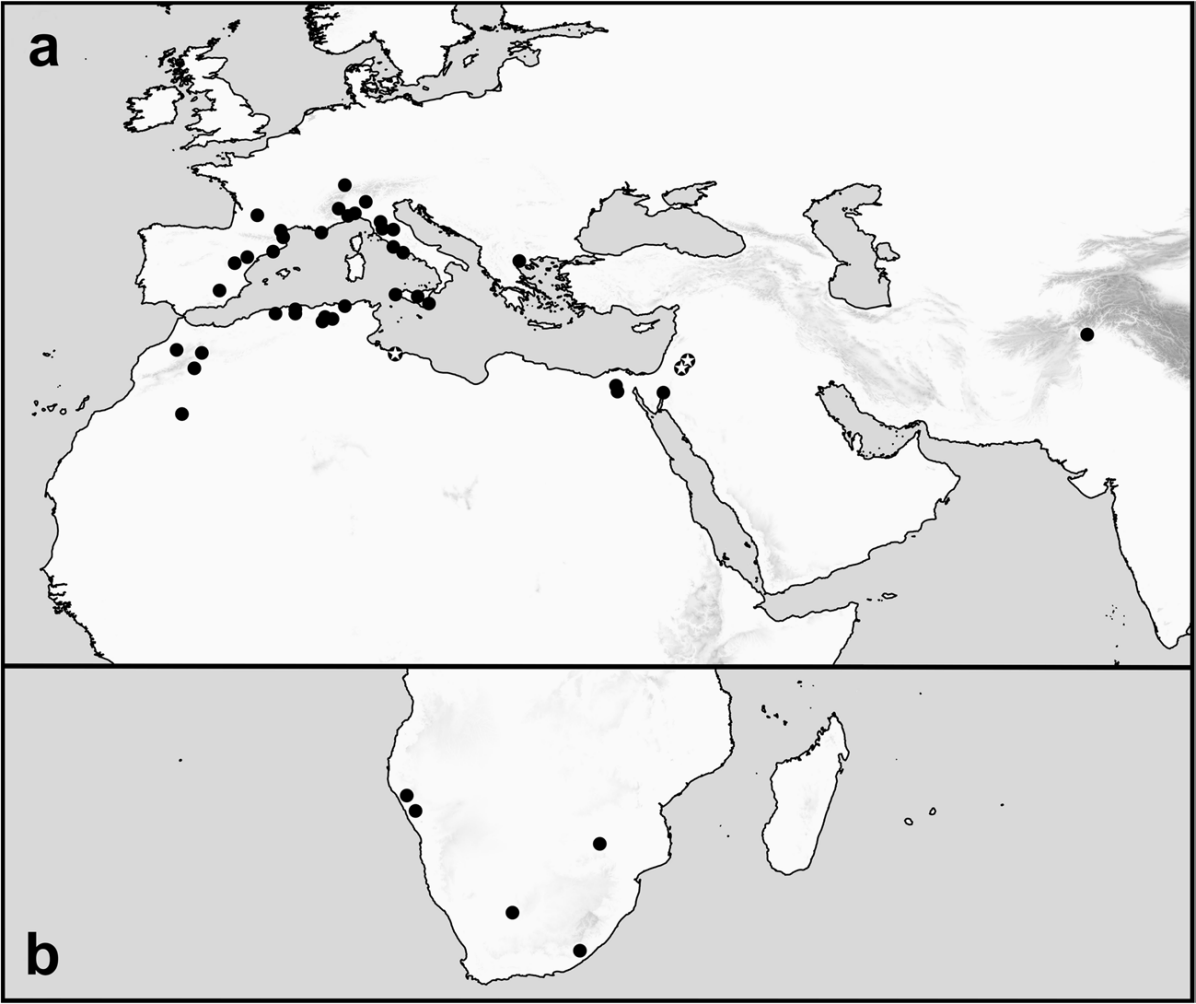
A map of the records of *Secretargas transgariepinus* in the south-western Palaearctic (**a**) and southern Africa (**b**). The records are reconstructed after currently known data (see Supplementary Data; circles); a circles with an asterisk mark the corrected records and new records of this species for the countries Libya and Jordan

The secretive bat-argas, *Secretargas transgariepinus* (White, 1846) (Ixodida: Argasidae), is a bat ectoparasite that occasionally parasitizes lizards and, rarely, humans (Reeves et al. 2020; Sándor et al. 2021). It belongs to the Afrotropical and Palearctic fauna, and its natural distribution is restricted to arid habitats of the subtropical zone. The geographical range of *S. transgariepinus* covers a belt of deserts and dry steppes extending from Morocco and southern Europe to Afghanistan in the Northern Hemisphere (Roshdy 1961; Sonenshine et al. 1962; Dusbábek 1970; Sándor et al. 2021). In the Southern Hemisphere, it occurs in South Africa and Namibia (White 1846; Neumann 1901, 1906; Howard 1908; Belford 1932, 1934; Pienaar et al. 2018; Hornok et al. 2019; Reeves et al. 2020). Within the northern section of the range, the northernmost records were from France and Switzerland where, however, the tick was considered to be imported by its migratory bat hosts (cf. Aeschlimann et al. 1965; Beaucournu 1966). The southernmost records in the Northern Hemisphere were from Algeria (Sándor et al. 2021). Most records of *S. transgariepinus* are from the western part of the Mediterranean Basin, in Morocco, Algeria, Spain, France, and Italy. In the central and eastern parts of the Mediterranean, abundant findings were reported only from the Cairo area of Egypt (Hoogstraal 1952) and, less frequently, from Greece and Israel (Hoogstraal 1952, 1957; Theodor and Costa 1960, 1967; Sándor et al. 2021; Fig. 1a, b; Table S1).

The rarity of records of *S. transgariepinus* is mainly due to its behavior. Indeed, like in most soft ticks, adults and nymphs parasitize their hosts for a very short time. The two stages are also hard to find because they quest around the bat roosting sites, hiding in small crevices and among rocks (cf. Berlese 1913; Hoogstraal 1952). Larvae have been collected more often, directly from their hosts, because they feed for longer periods. Larval specimens were mostly collected from bats of the genera *Hypsugo* Kolenati, 1856, *Plecotus* Geoffroy, 1818, and *Eptesicus* Rafinesque, 1820 (Mèdard et al. 1997; Medard et al. 2001). Collections from the environment (caves) represent a minority of records (White 1846; Methuen 1848; Hoogstraal 1957; Pienaar et al. 2018; Reeves et al. 2020.

Because *S. transgariepinus* can occasionally parasitize humans, it is important to establish if it can carry microorganisms of medical interest (Reeves et al. 2020; Sándor et al. 2021). *Secretargas transgariepinus* occurs in sympatry, and even syntopy, with another bat-associated soft tick, *Carios vespertilionis* (Latreille, 1802). In the Old World, *C. vespertilionis* is the soft tick species known to carry the largest number of microorganisms and, possibly, pathogens (Beaucournu and Clerck 1968; Sándor et al. 2021). The two tick species share hosts, and, in particular, Vespertilionidae, a bat family which has been found to be infected with a very diverse array of bacteria of the genera *Anaplasma* Theiler, 1910; *Bartonella* Strong, Tyzzer, Brues et Sellards, 1915; *Borrelia* Swellengrebel, 1907; *Coxiella* Philip, 1948; *Francisella* Dorofe’ev, 1947; *Leptospira* Noguchi, 1917; *Mycoplasma* Nowak, 1929; *Neorickettsia* Philip, Hadlow et Hughesand, 1953 and *Rickettsia* Da Rocha-Lima, 1916 (Szentiványi et al. 2023).

The first study of microbes in *S. transgariepinus* was based on materials from the Middle East, more precisely from caves in Ghiza, Egypt (leg. H. Hoogstraal). *Wolbachia* sp. Hertig, 1936, and/or *Rickettsia* were documented from the Malpighian tubules of this tick (Roshdy 1961, 1964). A total of 19 females, collected from one site in Ghiza, were used to test if they could transmit Keterah virus (KTRO, nairoviruses) (Varma and Converse 1976). Additionally, two microbes of unknown pathogenicity, *Rickettsia hoogstraalii*, a spotted fever group bacterium, and *Rickettsiella* sp., were detected with the help of molecular methods in *S. transgariepinus* from the Namib Desert of Namibia (Hornok et al. 2019; Reeves et al. 2020).

Recently, interest for Murid gammaherpesvirus 68 (MHV-68) has increased. Antibodies anti-MHV 68 and the DNA of the ORF50 of the MHV-68 virus were detected in domestic and wild mammals, including bats, but also in humans in Europe, Asia, and South America (Wágnerová et al. 2015; Briestenská et al. 2018; Janíková et al. 2020; Kabát et al. 2022). The MHV-68, a prototype strain of murid herpesvirus 4 (murid gammaherpesvirus 4, MuGHV4) from the genus *Rhadinovirus* (Herpesviridae) is closely related to the human oncogenic viruses: Epstein-Barr virus (human gammaherpesvirus 4, HuGHV4) from the genus *Lymphocryptovirus* and Kaposi’s sarcoma-associated virus (human gammaherpesvirus 8, HuGHV8) from the genus *Rhadinovirus* (Kaposi’s sarcoma-associated virus, the human gamma herpesvirus 8, HuGHV8), were first described by Dong et al. (2017) and Mistríková and Briestenská (2020). It is used as a murid laboratory model for a better understanding of the pathogenesis of similar human infections. In particular, studies have focused on revealing the mechanisms behind the development of malignancies such as Burkitt lymphoma, Hodgkin’s diseases, and/or Kaposi sarcoma (Dong et al. 2017; Mistríková and Briestenská 2020). The role of bats as reservoir host for this virus and the possible part played by ticks in transmitting it remain unexplored (Dietrich et al. 2016).

In this study, we re-determined specimens of soft ticks from Libya and Jordan that we suspected had been originally misidentified (see Saliba et al. 1990; Benda et al. 2010, 2014). We also screened 20 larval specimens from Jordan for the presence of tick-borne viral and bacterial microorganisms, where the presence of new pathogens for the mentioned species *Secretargas transgariepinus* was confirmed.

## Material and methods

### Study material

The examined material included one larval specimen found in a jar containing ticks from different bat species (four *Eptesicus isabellinus*, one *Myotis punicus*, and four *Pipistrellus kuhlii*) collected in Sabratha, Libya, on May 28, 2002, and deposited in the Zoological Collection of the National Museum, Prague, Czech Republic, leg. M. Ševčík. It had originally been identified as *Argas* sp. (see Benda et al. 2014: 130); 78 larvae, originally identified as *Argas vespertilionis* (Benda et al. 2010: 234), collected from a single female of *Otonycteris hemprichii* (NMP 92824) in the Shawmari Nature Reserve (SNR), Jordan, on July 10, 2010, leg. P. Benda and A. Reiter; and seven larvae, originally identified as *Ornithodoros salahi* (Saliba et al. 1990: 164) and collected from a bat determined as *Myotis* sp. (later identified as *Otonycteris hemprichii*, see Atallah (1967), Benda et al. (2010), and Ševčík et al. (2023)) of unidentified sex and age in Azraq-Shishan, Jordan, on May 2, 1966, leg. S. Atallah. For a description of the methods used to trap the bats and of other field records, refer to Saliba et al. (1990) and Benda et al. (2010, 2014).

### Morphological identification

The ticks were blot-dried on clean filter paper and observed under a stereomicroscope. The re-identification of the taxonomic affiliation of the ticks was carried out using morphological keys by Hoogstraal (1957: 546, Figs. 6–9; 548–549), Sonenshine et al. (1962: 205: Fig. 11, 208: Fig. 23 A, B), and Theodor and Costa (1960: 376, Text-Fig. 15–16, 377). The following key characters were used to distinguish *S. transgariepinus* from the argasid species at the source of the misidentifications: well-defined dorsal plate, with reticulate pattern consisting of convex and shining meshes; spiracular opening anterior to coxa II, relatively large and oval with numerous long setae projecting into its lumen; palps with fourth segments, the second segment significantly longer, almost as long as other three segments together; the fourth segment is much thinner than others (cf. Theodor and Costa 1960: 368, Fig. 4; 376–377, Fig. 16b).

Taxonomy and nomenclature of *Secretargas transgariepinus* follow the revision of the family Argasidae by Mans et al. (2021).

### Images

The images of the larva specimens of *S*. *transgariepinus* collected at Azraq-Shishan, Jordan, were taken by using the BK Plus Lab System (Visionary Digital), and stacked with Helicon Focus v. 4.77.

### Material depositories

The single larva from Sabratha, Libya, is deposited in the private collection of the first author (CMŠ [alcohol preparations]). Of the 78 larvae from the SNR, Jordan, originally housed at the Department of Zoology and Anthropology, Constantine the Philosopher University in Nitra, Slovakia (cf. Benda et al. 2014), 33 are currently deposited in the private collection of Martin Ševčík, Nitra, Slovakia (CMŠ [alcohol]); 25 in the collection of the National Museum, Prague, Czech Republic (NMP P6A 7529 [alcohol/withered preparations]); and 20 specimens in the collection of the Institute of Virology, Biomedical Research Center, Slovak Academy of Sciences, Bratislava, Slovakia (as a dissoluted DNA sample). The seven larvae from Azraq-Shishan, Jordan, leg. S. Atallah, are deposited in the US National Tick Collection, Statesboro, Georgia (USNMENT01786798).

### Pathogen screening and phylogenetic analyses

Twenty specimens from the material collected at the SNR, Jordan, were washed with 70% ethanol, then with sterile water, dried, transferred individually to tubes, and fragmented with a sterile Carbon Steel Surgical Scalpel Blade (Surgeon, JAI Surgicals Ltd., India). The DNA from the samples was isolated using the QIAamp DNA Mini Kit (Qiagen, Germany) according to the manufacturer’s instructions. The concentration and purity of the DNA were measured with a NanoPhotometer Pearl (Implen, Germany). The DNA samples were stored at − 20 °C and later used as templates for the PCR amplifications. Tick samples were tested for the presence of the MHV-68 virus by a nested PCR targeting the ORF50 gene (57) (Kabát et al. 2021; Table S2). Rickettsial organisms were first detected by real-time PCR with probe SFGP targeting RC0338 gene. Primers for *glt*A, *omp*A, and 17-kDa genes fragments (47, 54, 61) were then used to amplify the corresponding gene fragments by conventional PCR. The presence of bacteria *Anaplasma*/*Ehrlichia* spp., *Borrelia burgdorferi* sensu lato, *Bartonella* spp., and the piroplasms *Babesia* spp. (Regnery et al. 1991; Roux et al. 1996; Bekker et al. 2002; Derdáková et al. 2003; Courtney et al. 2004; Casati et al. 2006; Maggi et al. 2009; Socolovsch et al. 2010; Anstead and Chilton 2013; Table S2) were screened by conventional PCR (57, 60, 66, 55). The PCR amplicons were purified and analyzed by sequencing in both directions in Macrogen Inc. (Amsterdam, The Netherlands). The DNA sequences were compared with those available in GenBank using the Basic Local Alignment Search Tool (Blast; http://blast.ncbi.nlm.nih.gov). A phylogenetic tree was constructed using the Neighbor-Joining method (Saitou and Nei 1987). Evolutionary analyses were conducted in MEGA11 (Tamura et al. 2021). The evolutionary distances were computed using the p-distance method (Nei and Kumar 2000). A concatenated phylogenetic tree inferred from comparison of the *Rickettsia* 17-kDa, *gltA*, *ompA* partial sequences. Partial 17-kDa, *gltA* and *ompA* genes sequences for representative samples were submitted to the GenBank under the accession numbers OR900065–OR900068 for 17-kDa gene, OR900069–OR900071 for the *gltA* gene, and OR900072–OR900074 for the *ompA* gene.

## Results

### Records

**Fig. 2:**
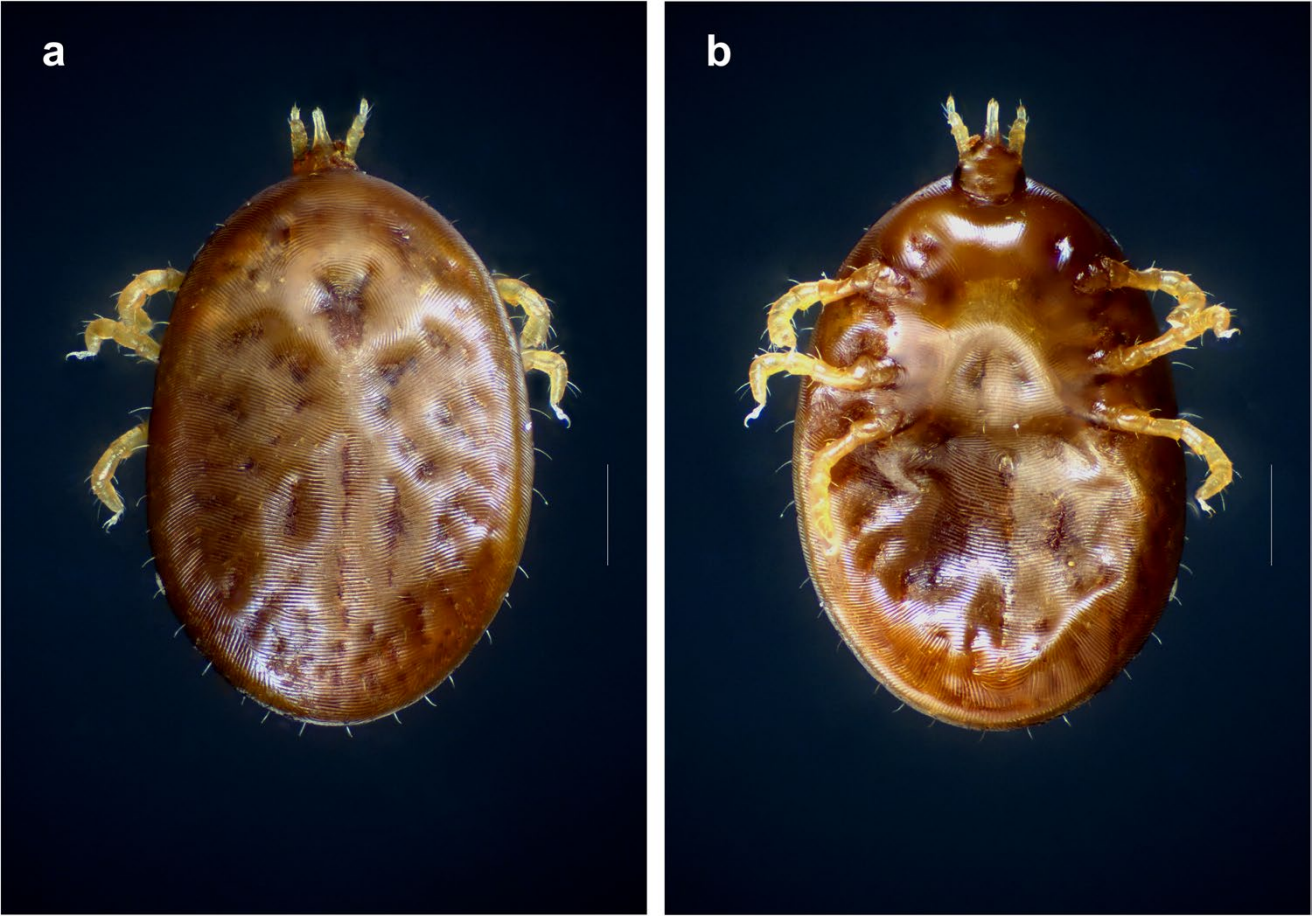
Engorged larva of *Secretargas transgariepinus* found on *Otonycteris hemprichii* at Azraq-Shishan, Jordan, originally identified as *Ornithodoros salahi* by Saliba et al. (1990). **a** Dorsal aspect. **b** Ventral aspect

The revision and comparison of the morphological characters of the examined specimens revealed that they all corresponded to *S. transgariepinus*. The engorged larva from Sabratha, Libya, was characterized by a typical dorsal plate and the respiratory system at the anterior surface of coxa 2, palps with fourth segments, the second segment significantly long, almost as long as other three segments, and the fourth segment is much thinner. Of the original eight engorged larvae from *Myotis* sp. (= *Otonycteris hemprichii*) from Azraq-Shishan, Jordan, seven specimens were left, of which only one was sufficiently well preserved for examination (see Fig. 2a, b). This specimen shared all relevant morphological characters with *S. transgariepinus*. In few of the 78 larvae (unengorged and engorged) from SNR, Jordan, the hypostome was broken and the spiracular plate was not visible. Otherwise, they also all corresponded to the mentioned description.

### Pathogens

Of the 20 samples of the *S. transgariepinus* analyzed by nested PCR, the presence of ORF50 sequence was confirmed in three of them. The obtained PCR product (580 bp) showed 100% homology to the sequence of the MHV68 WUMS strain (Acc No U97553.2) of the ORF50 gene (position from 68,219 to 68,799 nucleotides).

**Fig. 3:**
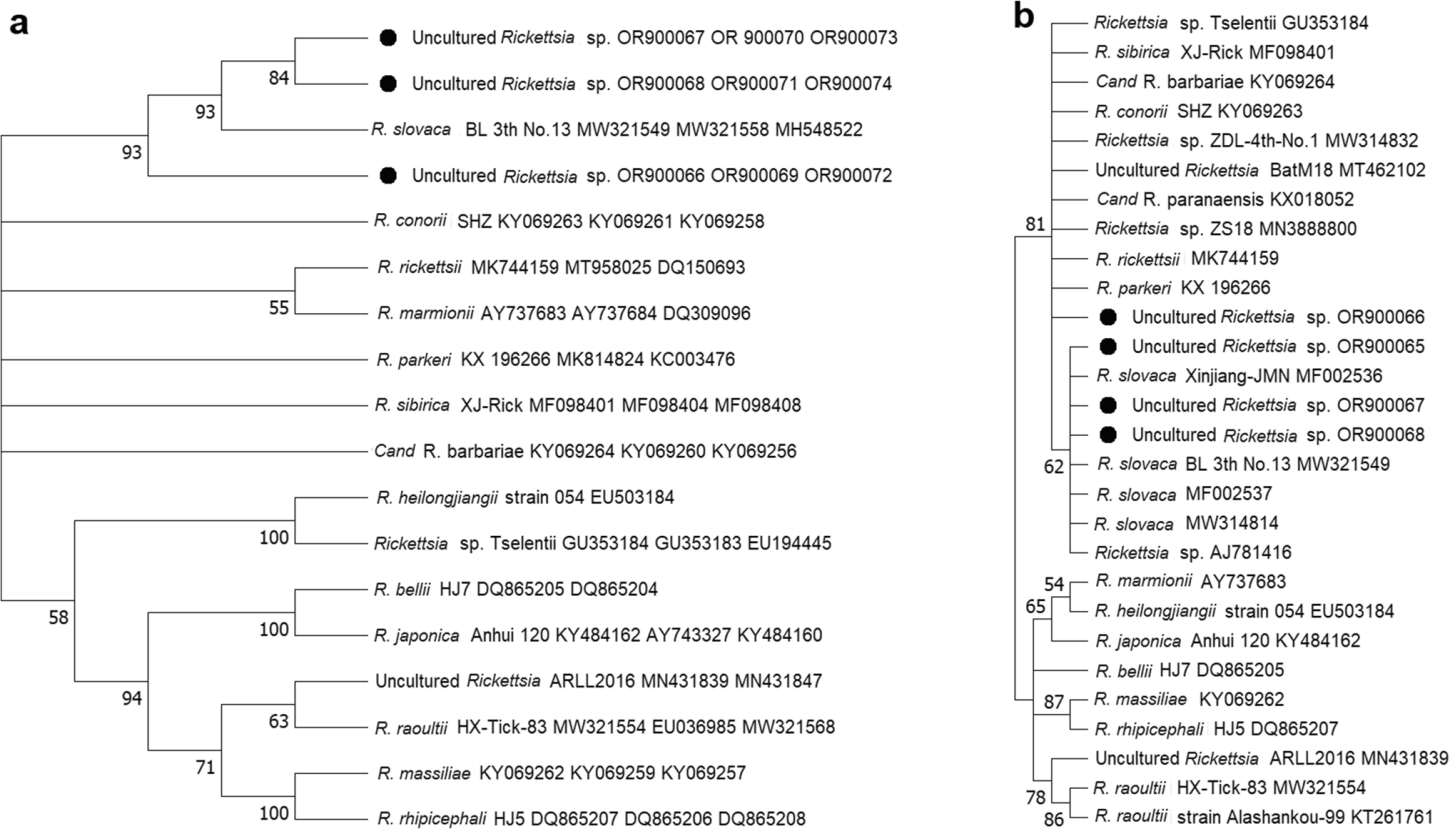
**a** A concatenated phylogenetic tree inferred from comparison of the *Rickettsia* 17-kDa, *gltA*, *ompA* partial sequences including those from *Secretargas transgariepinus* from the Shawmari Nature Reserve (SNR), Jordan, using the neighbor-joining method. **b** A tree inferred from partial sequences of the *Rickettsia* 17-kDa gene including those isolated from *Secretargas transgariepinus* from the SNR, Jordan, using the neighbor-joining method. The percentage of replicate trees in which the associated taxa clustered together in the bootstrap test (1000 replicates) are shown next to the branches (Felsenstein et al. 1985). GenBank accession numbers are included

One tick sample was “suspect positive” for *B. burgdorferi* sensu lato by a real-time PCR assay. However, because the *ct* value was > 36, the amplicon could not be successfully sequenced. As for rickettsial testing, while nine tick were positive by real-time PCR with probe SFGP targeting RC0338, only for three of them the amplification of *glt*A, *omp*A, and 17-kDa gene fragments was successful. In the phylogenetic reconstruction, the *S. transgariepinus* rickettsial lineages clustered with *R. slovaca* with a branch support of 96–98% (Fig. 3a). This was also confirmed for a fourth specimen for which only the 17-kDa gene fragment could be obtained (99.72%; 353/354 bp identity). A comparison of identities of the obtained sequences from *S*. *transgariepinus* larvae with sequences in GenBank is presented in Fig. 3b and Table S3.

## Discussion

### Correction of findings

The presence of the desert long-eared bat, *O. hemprichii*, among the hosts of the revised tick material suggested that some of the previously listed species could have been incorrectly identified. For the same reason, we questioned the original identification of the ticks.

*Otonycteris hemprichii* is a desert-dwelling species, distributed in arid areas of North Africa and the Middle East (Benda and Gvoždík 2010). It roosts mainly in rock crevices and small cavities which can support the life cycle of a tick that likes to hide, such as *Argas confusus* Hoogstraal, 1955, or *S*. *transgariepinus* (see Hoogstraal 1955).

The oldest record of *S*. *transgariepinus* from the Middle East, eight larvae from Azraq-Shishan, Jordan, was originally referred to as *Ornithodoros* (*Reticulinasus*) *salahi* by Saliba et al. (1990). Ševčík et al. (2023: 1274, 1277) reidentified the host species as *O. hemprichii*, instead of the originally reported *Myotis* sp. (see also Atallah 1967 and Benda et al. 2010). This reidentification of the host species, which favors harsh deserts, along with the long distance of Azraq-Shishan from the range of the Egyptian fruit bat, *Rousettus agyptiacus*, a primary host of *Reticulinasus salahi* (more than 100 km to the Rift Valley in western Jordan, see Benda et al. 2010) suggested a possible concomitant misidentification of the tick species. Indeed, *S. transgariepinus* seemed to be the most probable candidate for the correct identification, as was confirmed by a detailed morphological re-examination of one of the specimens mentioned by Saliba et al. (1990).

The second record from Jordan was originally described as follows: “*Argas vespertilionis*: 78 larvae (UCPN) from 1 fa (NMP 92824), Shawmari Wildlife Reserve, July 10, 2010, from host *Otonycteris hemprichii*” (Benda et al. 2010: 294). The unusually large number of parasites observed on a single bat was documented by a photograph showing a very heavy tick infestation (Benda et al. 2010: 291, Fig. 83). The hypothesis that these ticks that were also incorrectly identified was supported by our re-examination: all these ticks were in fact *S. transgariepinus*.

The last record comes from Libya; a single specimen was found in a museum jar containing three species of bats (Benda et al. 2014: 130) as follows: “*Argas* sp. (*A*. *vespertilionis* group): one larva (CMŠ [P]) from a jar containing four [specimens of] *Eptesicus isabellinus*, one *Myotis punicus* and four *Pipistrellus kuhlii*, Sabratha, May 28, 2002.” The morphological examination of the concerned specimens led to them being reassigned to *S*. *transgariepinus*. Sándor et al. (2021) suggested a possible occurrence of this tick species in the western part of Libya, an area included in the distribution range of its main bat hosts, *E. isabellinus* (Temminck, 1840); *Plecotus gaisleri* Benda, Kiefer, Hanák et Veith, 2004; and *Hypsugo savii* (Bonaparte, 1837). As *H. savii* does not occur in Libya (Benda et al. 2014), we can assume that the tick most probably originates from *E*. *isabellinus* or *P*. *gaisleri*.

However, the group of main hosts of this tick most probably covers a broad spectrum of bat species and consequently; also, its distribution range is probably much larger than currently known. The above mentioned host, *O. hemprichii*, is referred by Sándor et al. (2021) as a secondary host species. However, our evidence would indicate that it as one of the primary hosts of *S*. *transgariepinus*. The bats of the genus *Plecotus*, of which three species live in Africa and use a similar roosting strategy, have also been described as primary hosts of *S*. *transgariepinus*. It is important to mention that ticks, in general, have often been found to be more dependent for their survival on the availability of suitable environments (in this case secluded rock crevices) than on the presence of so-called specific/primary/secondary hosts (Klompen et al. 1996). The available data confirmed the occurrence of this tick also in the tropics of Africa (from *Plecotus balensis* Kruskop et Lavrenchenko, 2000, Desea Forest, Ethiopia, 13°53′N, 39°46′E, October 30, 2012, leg. P. Benda, own unpubl. data).

As a result, the traditionally treated ecological preferences of *S*. *transgariepinus* have to be re-defined. Originally thought to require very arid environments, such as those encountered in Egypt and the Northern Cape province of South Africa (Hoogstraal 1952; Pienaar et al. 2018), it is now clear that the species occurs also in more humid areas.

### Murid gammaherpesvirus 4 (MHV-68), prototype strain

With the help of the PCR assays, we confirmed the presence of the MHV-68 virus in four of 20 larva specimens of *Secretargas trangariepinus* collected from a single host specimen of *O. hemprichii* at the SNR, Jordan. The combination of the available data, i.e., collection of ticks from a single host bat individual, the size of the examined sample set (20 samples), and the method used (PCR) do not allow us to speculate on the vectorial capacity of the ticks. Indeed, a virus circulating in the bat blood would be ingested by the ticks (and detected by PCR in the tick DNA samples) even if the ticks might not be able to further transmit the virus to other hosts. Our results, like those of other studies involving bats and ectoparasites (Briestenská et al. 2018; Janíková et al. 2020; Ševčík et al. 2023), cannot be interpreted for the time being. Laboratory transmission experiments will be required to elucidate whether or not *S. transgariepinus* ticks are competent vectors of MHV-68. Nevertheless, this represents the second geographical record of occurrence of this virus in the Middle East, after its discovery in *Reticulinasus salahi* in the Al Hotta Cave, Oman (Ševčík et al. 2023).

### Bacteria

The positivity for *Borrelia burgdorferi* s.l. was confirmed in a single larva of *S. trangariepinus* from the SNR, Jordan, by real-time PCR. The reservoir competence of various vertebrate species for *B*. *burgdorferi* s.l. is determined by their capacity to effectively infect pathogen-free larval ticks under natural conditions or in xenodiagnostic experiments (Mannelli et al. 2012). The abundance of *B. burgdorferi* s.l. in the larvae of chiropterophilous hard ticks of the genus *Ixodes* predicts that the vespertilionid bats are most probably the reservoir hosts and effective vectors of this bacterium (Michalik et al. 2020). While our results prove the occurrence of a *B. burgdorferi* s.l. genotype in *S. transgariepinus*, they will have to be corroborated by additional studies and, more importantly, the detected spirochete will have to be fully characterized in order to gain any kind of understanding of its epidemiological importance (cf. Obaidat et al. 2020). So far, very little is known about the ecoepidemiology of borrelioses in the Middle East (records are known from Israel and Turkey; Abraham et al. 1991; Polat et al. 1998; Guner et al. 2003). Even less is known about the genetic diversity of *Borrelia* sp. in this area.

The *gltA*, *ompA*, and 17-kDa gene sequences amplified and sequenced from three samples of Jordanian larvae showed that the rickettsial organism in *S. transgariepinus* is a close relative of *R. slovaca*, a member of the spotted fever group (SFG) that was initially isolated in 1968 from the tick *Dermacentor marginatus* Sulzer, 1776, in Slovakia (Rehacek 1984). Although the presence of this pathogen and its role in bats is not known, in humans it can cause tick-borne lymphadenopathy (TIBOLA), also called *Dermacentor*-borne necrosis erythema and lymphadenopathy (DEBONEL) (Lakos 1997; Oteo et al. 2004). The available records of *R*. *slovaca* are linked to its main vector, *D*. *marginatus*, but also to other tick species from southern and central parts of Europe (Rehacek 1984, Beati et al. 1993, 1994; Selmi et al. 2008; Raoult et al. 2002; Špitalská et al. 2012) and, less so, from northern Africa and Asia (Shpynov et al. 2006; Sarih et al. 2008; Jiang et al. 2012; Kernif et al. 2012; Tian et al. 2012, Piotrowski and Rymaszewska 2020). It is interesting to note that *D. marginatus* has sporadically been collected from bats, for instance from *Pipistrellus pipistrellus* (Schreber, 1774) in Iran (Filippova et al. 1976), from *Myotis blythii* (Tomes, 1857), and *Rhinolophus euryale* Blasius, 1853 in Azerbaijan (Gadžiev and Dubovčenko 1975; Gadžiev et al. 1990). Although rare, these findings can explain how *R. slovaca* might have been introduced into bats. Recently, *R. slovaca* was detected in the visceral organs of Asian bats (Zhao et al. 2020) providing additional support for an epidemiological relationship between this specific rickettsial pathogen and bats. All our records can show, for the time being, that the bacterium can be found in engorged *S. transgariepinus* and that its distribution range now reaches Jordan.

### Supplementary Information

Below is the link to the electronic supplementary material.

Supplementary file1 (XLSX 16.1 KB)

Supplementary file2 (DOCX 22 KB)

## Data Availability

No datasets were generated or analysed during the current study.

## References

[CR1] Abraham Z, Feuerman EJ, Rozenbaum M, Glück Z (1991) Lyme disease in Israel. J Amer Acad Dermatol 25:729. 10.1016/S0190-9622(08)80681-0 10.1016/s0190-9622(08)80681-0 1791231

[CR2] Aeschlimann A, Büttiker W, Elbl A, Hoogstraal H (1965). A propos des Tiques de Suisse (Arachnoidea, Acarina, Ixodoidea). Rev Suisse Zool.

[CR3] Anstead CA, Chilton NB (2013). A novel Rickettsia species detected in vole ticks (Ixodes angustus) from western Canada. Appl Environ Microbiol.

[CR4] Atallah SI (1967) Mammalogy (with a list of reptiles and amphibians). In: Boyd JE (ed) International Jordan Expedition 1966, Unpublished report, International Biological Programme, Conservation of Terrestrial Communities Section, London, pp 56–63

[CR5] Beati L, Finidori JP, Raoult D (1993). First isolation of Rickettsia slovaca from Dermacentor marginatus in France. Am J Trop Med Hyg.

[CR6] Beati L, Humair PF, Aeschlimann A, Raoult D (1994). Identification of spotted fever group rickettsiae isolated from Dermacentor marginatus and Ixodes ricinus ticks collected in Switzerland. Am J Trop Med Hyg.

[CR7] Beaucournu J-C (1966). Sur quelques Ixodoidea (Acarina) paléarctiques inféodés aux micro-Chiroptères. Ann Par.

[CR8] Beaucournu JC, Clerc B (1968). Argas (Secretargas) transgariepinus White, 1846, tique nouvelle pour la France et l’Algerie. Vie Et Millieu.

[CR9] Bedford GAH (1932). A synoptic check-list and host-list of the ectoparasites found in South African Mammalia, Aves and Reptilia. Rep Dir Vet Ser an Ind S Afr.

[CR10] Bedford GAH (1934). South African Ticks. Part I. Onderst J Vet Sci Anim Ind.

[CR11] Bekker CPJ, de Vos S, Taoufik A, Sparagano OAE, Jongejan F (2002) Simultaneous detection of *Anaplasma* and *Ehrlichia* spp. in ruminants and detection of *Ehrlichia ruminantium* in *Amblyomma variegatum* ticks by reverse line blot hybridization.Vet Microbiol 89:223–238. 10.1016/S0378-1135(02)00179-7 10.1016/s0378-1135(02)00179-7 12243899

[CR12] Benda P, Gvoždík V (2010). Taxonomy of the genus Otonycteris (Chiroptera: Vespertilionidae: Plecotini) as inferred from morphological and mtDNA Data. Acta Chiropterol.

[CR13] Benda P, Lučan RK, Obuch J, Reiter A, Andreas M, Bačkor P, Bohnenstengel T, Eid EK, Ševčík M, Vallo P, Amr ZS (2010). Bats (Mammalia: Chiroptera) of the Eastern Mediterranean and Middle East. Part 8. Bats of Jordan: fauna, ecology, echolocation, ectoparasites. Acta Soc Zool Bohem.

[CR14] Benda P, Spitzenberger F, Hanák V, Andreas M, Reiter A, Ševčík M, Šmíd J, Uhrin M (2014). Bats (Mammalia: Chiroptera) of the Eastern Mediterranean and Middle East. Part 11. On the bat fauna of Libya II. Acta Soc Zool Bohem.

[CR15] Berlese A (1913). Sopra una specie di *Argas* nuova per lʼItalia. Redia.

[CR16] Briestenská K, Janíková M, Kabát P, Csepányiová D, Zukal J, Pikula J, Kováčová V, Linhart P, Banďouchová H, Mistríková J (2018). Bats as another potential source of murine gamma herpesvirus 68 (MHV-68) in nature. Acta Virol.

[CR17] Casati S, Sager H, Gern L, Piffaretti J-C (2006). Presence of potentially pathogenic *Babesia* sp. for human in *Ixodes ricinus* in Switzerland. Ann Agric Environ Med.

[CR18] Courtney JW, Kostelnik LM, Zeidner NS, Massung RF (2004). Multiplexreal-time PCR for detection of Anaplasma phagocytophilum and Borrelia burgdorferi. J Clin Microbiol.

[CR19] Derdáková M, Beati L, Peťko B, Stanko M, Fish D (2003). Genetic variability within Borrelia burgdorferi sensu lato genospecies established by PCR-single-strand conformation polymorphism analysis of the rrfA-rrlB intergenic spacer in Ixodes ricinus ticks from the Czech Republic. Appl Environ Microbiol.

[CR20] Dietrich M, Tjale MA, Weyer J, Kearney T, Seamark ECJ, Nel LH, Monadjem A, Markotter W (2016) Diversity of *Bartonella* and *Rickettsia* spp. in bats and their blood-feeding ectoparasites from South Africa and Swaziland. PLOS ONE 11(3): e0152077. 10.1371/journal.pone.0152077 10.1371/journal.pone.0152077 PMC4801393 26999518

[CR21] Dong S, Forrest JC, Liang X (2017). Murine gammaherpesvirus 68: a small animal model for gammaherpesvirus-associated diseases. Adv Exp Med Biol.

[CR22] Dusbábek F (1970). Mite parasites (Acarina) of bats from Afghanistan. Fol Parasit.

[CR23] Filippova NA, Neronov VM, Farhang-Azad A (1976) [Data on the ixodid fauna (Acarina, Ixodidae) of small mammals in Iran]. Entomologičeskoe Obozrenie 55:467–479 [Russian original not seen; Translation 1169 Medical Zoology Department United States Naval Medical Research Unit Number Three Cairo, Egypt]

[CR24] Gadžiev AT, Dubovčenko TA (1975). Ektoparazity ostrouhoj nočnicy [Ectoparasites of the lesser mouse-eared bat]. Probl Parazitol.

[CR25] Gadžiev AT, Dubovčenko TA, Džafarov GD (1990) Sostav fauny ektoparazitov podkovonosov (*Rhinolophus*) na territorii SSSR [Composition of the ectoparasite fauna of the horseshoe bats (*Rhinolophus*) in the territory of the Soviet Union]. In: Il’in VJ, Strelkov PP, Rodinov VA (eds) Rukokrylye: Materialy Pâtogo Vsesoûznogo Soveŝaniâ po Rukokrylym (Chiroptera) [Bats: Proceedings of the Fifth Pan-Union Conference on Bats], Vsesoûznoe Teriologičeskoe Obŝestvo & Penzenskij Gosudarstvennyj Pedagogičeskij Institut Imeni V. G. Belinskogo, Penza, pp 122–129 [in Russian]

[CR26] Guner ES, Hashimoto N, Takada N, Kaneda K, Imai Y, Masuzawa T (2003) First isolation and characterization of *Borrelia burgdorferi* sensu lato strains from *Ixodes ricinus* ticks in Turkey. J Med Microbiol 52:807–813.10.1099/jmm.0.05205-0 10.1099/jmm.0.05205-0 12909659

[CR27] Hoogstraal H (1952). Note on Egyptian ticks. I. — the genus *Argas* in the Cairo area. Proc Egypt Acad Sci.

[CR28] Hoogstraal H (1957) Bat ticks of the genus *Argas* (Ixodoidea, Argasidae) 2. *Secretargas* new subgenus and *A*. *transgariepinus* White, 1846, its adult and immature stages; with a definition of the subgenus *Argas*. Ann Entomol Soc Am 50:544–549. 10.1093/aesa/50.6.544

[CR29] Hornok S, Szoke K, Meli ML, Sándor AD, Görföl T, Estók P, Wang Y, Tu VT, Kováts D, Boldogh SA, Corduneanu A, Sulyok KM, Gyuranecz M, Kontschán J, Takács N, Halajian A, Epis S, Hofmann-Lehmann R (2019). Molecular detection of vector-borne bacteria in bat ticks (Acari: Ixodidae, Argasidae) from eight countries of the Old and New Worlds. Parasit Vectors.

[CR30] Howard CW (1908) A list of the ticks of South Africa, with descriptions and keys to all the forms known. Ann Transv Mus l:73–172

[CR31] Janíková M, Briestenská K, Salinas-Ramos VB, Mistríková J, Kabát P (2020). Molecular detection of murine gammaherpesvirus 68 (MHV-68) in bats from Mexico. Acta Virol.

[CR32] Jiang J, You BJ, Liu E, Apte A, Yarina TR, Myers TE, Lee JS, Francesconi SC, O’Guinn ML, Tsertsvadze N, Vephkhvadze N, Babuadze G, Sidamonidze K, Kokhreidze M, Donduashvili M, Onashvili T, Ismayilov A, Agayev N, Aliyev M, Muttalibov N, Richards AL (2012). Development of three quantitative real-time PCR assays for the detection of Rickettsia raoultii, Rickettsia slovaca, and Rickettsia aeschlimannii and their validation with ticks from the country of Georgia and the Republic of Azerbaijan. Ticks Tick Borne Dis.

[CR33] Kabát P, Briestenská K, Ivančová M, Trnka A, Špitalská E, Mistríková J (2021). Birds belonging to the family Paridae as another potential reservoir of murine gammaherpesvirus 68. Vect Born Zoon Dis.

[CR34] Kabát P, Hricková N, Ivančová M, Jablonski D, Briestenská K, Bohuš M, Krajanová V, Mistríková J (2022). Ectotherm vertebrates as a new potential reservoir of murid gammaherpesvirus 4. Acta Virol.

[CR35] Kernif T, Messaoudene D, Ouahioune S, Parola P, Raoult D, Bitam I (2012). Spotted fever group rickettsiae identified in Dermacentor marginatus and Ixodes ricinus ticks in Algeria. Ticks Tick Borne Dis.

[CR36] Klompen JSH, Black WC, Keirans JE, Oliver JH (1996). Evolution of Ticks Ann Rev Ent.

[CR37] Lakos A (1997). Tick-borne lymphadenopathy—a new rickettsial disease?. Lancet.

[CR38] Maggi RG, Kosoy M, Mintzer M, Breitschwerdt EB (2009). Isolation of candidatus Bartonella melophagi from human blood. Emerg Infect Dis.

[CR39] Mannelli A, Bertolotti L, Gern L, Gray J (2012). Ecology of Borrelia burgdorferi sensu lato in Europe: transmission dynamics in multi-host systems, influence of molecular processes and effects of climate change. FEMS Microbiol Rev.

[CR40] Mans BJ, Kelava S, Pienaar R, Featherston J, de Castro MH, Quetglas J, Reewes WK, Durden LA, Miller MM, Laverty TM, Shao R, Takano A, Kawabata H, Moustafa MAM, Nakao R, Matsuno K, Greay TL, Evasco KL, Barker D, Barker SC (2021). Nuclear (18S–28S rRNA) and mitochondrial genome markers of *Carios* (*Carios*) *vespertilionis* (Argasidae) support *Carios* Latreille, 1796 as a lineage embedded in the Ornithodorinae: re-classification of the *Carios* sensu Klompen and Oliver (1993) clade into its respective subgenera. Ticks Tick Borne Dis.

[CR41] Mèdard P, Guiguen C, Beaucournu J-C (1997). Nouvelles recoltes d’Argas transgariepinus White, 1846 tique de chiropteres (Acarina–Ixodoidea–Argasidae) en France et au Maroc [New data on Argas transgariepinus White, 1846, the bat ticks (Acarina – Ixodoidea – Argasidae), in France and Morocco]. BIPAS.

[CR42] Medard P, Guiguen C, BeaucournuJC, (2001). Nouvelle récoltes d’
* Argas transgariepinus
* White, 1846, tique de chiroptères (Acarina – Ixodoidea – Argasidae) en France et au Maroc. Sci Rep Port-Crosnatl Park. Fr; Notes Brèves.

[CR43] Methuen HH (1848) Life in the wilderness; or wanderings in South Africa. Richard Bentley, London

[CR44] Michalik J, Wodecka B, Liberska J, Dabert M, Postawa T, Piksa K, Stańczak J (2020). Diversity of *Borrelia burgdorferi* sensu lato species in *Ixodes* ticks (Acari: Ixodidae) associated with cave-dwelling bats from Poland and Romania. Ticks Tick Borne Dis.

[CR45] Mistríková J, Briestenská K (2020). Muridherpesvirus 4 (MuHV-4, prototype strain MHV-68) as an important model in global research of human oncogenic gamma herpesviruses. Acta Virol.

[CR46] Nei M, Kumar S (2000). Molecular evolution and phylogenetics.

[CR47] Neumann LG (1901). Revision de la famille des ixodides. 4e mémoire. Mem Soc Zool Fr.

[CR48] Neumann LG (1906). Notes sur les ixodides, IV. Arch Parasit.

[CR49] Obaidat MM, Alshehabat MA, Hayajneh WA, Roess AA (2020). Seroprevalence, spatial distribution and risk factors of *Borrelia burgdorferi* sensu lato in Jordan. Comp Immun, Microb Inf Dis.

[CR50] Oteo JA, Ibarra V, Blanco JR, Martínez de Artola V, Márquez FJ, Portillo A, Raoult D, Anda P (2004). *Dermacentor*-borne necrosis erythema and lymphadenopathy: clinical and epidemiological features of a new tick-borne disease. Clin Microbiol Infect.

[CR51] Pienaar R, de Klerk DG, Putterill JF, Mans BJ (2018). Notes on maternal behaviour in soft ticks: specifically observed in Argas (Argas) striatus Bedford, 1932 and Argas (Secretargas) transgariepinus White, 1846. Ticks Tick Borne Dis.

[CR52] Piotrowski M, Rymaszewska A (2020). Expansion of tick-borne rickettsioses in the world. Microorganisms.

[CR53] Polat E, Calisir B, Yucel A, Tuzer E (1998). Türkiye’de *Ixodes ricinus*’ lardan ilk defa ayrılan ve üretilen iki *Borrelia kökeni*. Turkiye Parazitol Derg.

[CR54] Raoult D, Lakos A, Fenollar F, Beytout J, Brouqui P, Fournier PE (2002). Spotless rickettsiosis caused by *Rickettsia slovaca* and associated with *Dermacentor* ticks. Clin Infect Dis.

[CR55] Reeves WK, Mans BJ, Durden LA, Miller MM, Gratton EM, Laverty TM (2020). *Rickettsia hoogstraalii* and a *Rickettsiella* from the bat tick *Argas transgariepinus*, in Namibia. J Parasitol.

[CR56] Regnery RL, Spruill CL, Plikaytis BD (1991). Genotypic identification of rickettsiae and estimation of intraspecies sequence divergence for portions of two rickettsial genes. J Bacteriol.

[CR57] Rehácek J (1984). *Rickettsia slovaca*, the organism and its ecology [review]. Prir Prac Ustav Ceskoslov Akad Ved Brne.

[CR58] Roshdy MA (1961). Intracellular *Rickettsia*-like micro-organisms in certain ticks. Nature.

[CR59] Roshdy MA (1964). Rickettsia like microorganism in the malpighian tubules and ovary of argas boueti roubaud and colas-belcour, a. vespertilionis latreille, a. transgariepinus white and a. brumpti neumann. Proc Egypt Acad Sciu.

[CR60] Roux V, Fournier PE, Raoult D (1996). Differentiation of spotted fever group rickettsiae by sequencing and analysis of restriction fragment length polymorphism of PCR-amplified DNA of the gene encoding the protein rOmpA. J Clin Microbiol.

[CR61] Saitou N, Nei M (1987). The neighbor-joining method: a new method for reconstructing phylogenetic trees. Mol Biol Evol.

[CR62] Saliba EK, Amr ZS, Wassef HY, Hoogstraal H, Main AJ (1990). The ticks (Ixodoidea) of East Jordan and the West Bank. Dirasat S B.

[CR63] Sándor AD, Mihalca AD, Domşa C, Péter Á, Hornok S (2021). Argasid ticks of Palearctic bats: distribution, hostselection, and zoonoticimportance. Front Vet Sci.

[CR64] Sarih M, Socolovschi C, Boudebouch N, Hassar M, Raoult D, Parola P (2008). Spotted fever group rickettsiae in ticks, Morocco. Emerg Infect Dis.

[CR65] Selmi M, Bertolotti L, Tomassone L, Mannelli A (2008). *Rickettsia slovaca* in *Dermacentor marginatus* and tick-borne lymphadenopathy, Tuscany. Italy Emerg Infect Dis.

[CR66] Ševčík M, Špitalská E, Kabát P, Lučan RK, Maliterná M, Reiter A, Uhrin M, Benda P (2023). *Reticulinasus salahi* (Acarina: Argasidae), a tick of bats and man in the Palaearctic and Afrotropics: review of records with the first pathogens detected. Parasitol Res.

[CR67] Shpynov SN, Fournier PE, Rudakov NV, Samoilenko IE, Reshetnikova TA, Yastrebov VK, Schaiman MS, Tarasevich IV, Raoult D (2006). Molecular identification of a collection of spotted fever group rickettsiae obtained from patients and ticks from Russia. Am J Trop Med Hyg.

[CR68] Socolovsch C, Mediannikov O, Sokhna C, Tall A, Diatta G, Bassene H, Trape JF, Raoult D (2010). *Rickettsia felis*-associated uneruptive fever, Senegal. Emerg Infect Dis.

[CR69] Sonenshine DE, Clifford GM, Kohls GM (1962). The identification of larvae of the genus
* Argas
* (Acarina: Argasidae). Acarologia.

[CR70] Špitalská E, Štefanidesová K, Kocianová E, Boldiš V (2012). *Rickettsia slovaca* and *Rickettsia raoultii* in *Dermacentor marginatus* and *Dermacentor reticulatus* ticks from Slovak Republic. Exp Appl Acarol.

[CR71] Szentiványi T, McKee C, Jones G, Foster JT (2023) Trends in bacterial pathogens of bats: global distribution and knowledge gaps. Trans Emer Dis 9285855. 10.1155/2023/9285855 10.1155/2023/9285855 PMC12017137 40303798

[CR72] Tamura K, Stecher G, Kumar S (2021). MEGA 11: Molecular Evolutionary Genetics Analysis Version 11. Mol Biol Evol.

[CR73] Theodor O, Costa M (1967) A survey of the parasites of wild mammals and birds in Israel. Part one. Ectoparasites. Israel Academy of Sciences and Humanities, Jerusalem

[CR74] Theodor O, Costa M (1960). New species and new records of Argasidae from Israel. Observations on the rudimentary scutum and the respiratory system of the larvae of theArgasidae. Parasitology.

[CR75] Tian ZC, Liu GY, Shen H, Xie JR, Luo J, Tian MY (2012). First report on the occurrence of Rickettsia slovaca and Rickettsia raoultii in Dermacentor silvarum in China. Parasites Vectors.

[CR76] Varma MGR, Converse JD (1976). Keterah virus infections in four species of Argas ticks (Ixodoidea: Argasidae). J Med Entomol.

[CR77] Wágnerová M, Chalupková A, Hrabovská Z, Ančicová L, Mistríková J (2015). Possible role of different animal species in maintenance and spread of murine gammaherpesvirus 68 in the nature. Acta Virol.

[CR78] White A (1846) List of Annulosa, (principally insects) found on the journey of Henry H. Methuen, Esq. In: Methuen (ed) Life in the Wilderness; or Wanderings in South Africa, Richard Bentley, London, pp 307–318

[CR79] Zhao S, Yang M, Liu G, Hornok S, Zhao S, Sang C, Tan W, Wang Y (2020). Rickettsiae in the common pipistrelle Pipistrellus pipistrellus (Chiroptera: Vespertilionidae) and the bat soft tick Argas vespertilionis (Ixodida: Argasidae). ParasitesVectors.

