# Supplementary material for: First records of Secretargas transgariepinus (Argasidae) in Libya and Jordan: corrections of collection records and detection of microorganisms
Source: Parasitol Res. 2024 May 28;123(5):223. doi: 10.1007/s00436-024-08239-5 (PMC11133098; doi:10.1007/s00436-024-08239-5)
Supplement: Supplementary file 2 — Supplementary file2 (DOCX 22 KB) [file 436_2024_8239_MOESM2_ESM.docx]

**Supplementary material**

**Table 2** Primers used for the detection and/or identification of viruses and diferent vector-borne bacteria in the examined samples of *Secretargas transgariepinus* from Jordan

| Assay (viruses and bacteria) | Primer name | Primer sequence (5´- 3´) | Target gene | A. g. (bp) | A. t. (°C) | Reference |
| --- | --- | --- | --- | --- | --- | --- |
| PCR (viruses) |  |  |  |  |  |  |
| MHV–68 | ORF50 F1 | CCACCTGATCAAATATGCCA | ORF50 gene of MHV-68 | 969 | 57 | Kabát et al. (2021) |
|  | ORF50 R1 | TGTGGGTTTCTTGTTTGGAC | ORF50 gene of MHV-68 |  |  |  |
|  | ORF50 F2 | TGGCATATCCAGAGAAGTTGAG | ORF50 gene of MHV-68 | 581 | 57 |  |
|  | ORF50 R2 | TGGGAGTAGGTATGTAGCTCTG | ORF50 gene of MHV-68 |  |  |  |
| PCR (bacteria) |  |  |  |  |  |  |
| *Rickettsia* spp. | SFGF | GAM AAA TGA ATT ATA TAC GCC GCA AA | hypothetical protein (RC0338 gene) | 109 | 60 | Socolovsch et al. (2010) |
|  | SFGR | ATT ATT KCC AAA TAT TCG TCC TGT AC |  |  |  |  |
|  | SFGP | CTC AAG ATA AGT ATG AGT TAA ATG TAA A |  |  |  |  |
|  | RpCs.877p | GGG GGC CTG CTC ACG GCG G | citrate synthase (gltA) gene | 380 | 47 | Regnery et al. (1991) |
|  | RpCs.1258n | ATT GCA AAA AGT ACA GTG AAC A |  |  |  |  |
|  | Rr190.70p | ATG GCG AAT ATT TCT CCA AAA | outer membrane protein A (ompA) gene | 632 | 54 | Roux et al. (1996) |
|  | RR190.701R | GTT CCG TTA ATG GCA GCA TCT |  |  |  |  |
|  | 17K-5 | GCT TTA CAA AAT TCT AAA AAC CAT ATA | 17-kDa antigen gene | 434 | 61 |  |
|  | 17K-3 | TGT CTA TCA ATT CAC AAC TTG CC |  |  |  |  |
|  | 17kD1 | GCT CTT GCA ACT TCT ATG TT |  | 434 | 61 | Anstead and Chilton (2013) |
|  | 17kD2 | CAT TGT TCG TCA GGT TGG CG |  |  |  |  |
| PCR |  |  |  |  |  |  |
| *Anaplasma*/*Ehrlichia* spp. | 16S8FE | AGA GTT KGA TCM TGG YTC AG | 16rRNA gene spanning the V1 region | 470 | 57 | Bekker et al. (2002) |
|  | B-GA1B | CGA GTT TGC CGG GAC TTY TTC T | 16rRNA gene spanning the V1 region |  |  |  |
| PCR |  |  |  |  |  |  |
| *Borrelia burgdorferi* sensu lato | Bb23Sf | CGAGTCTTAAAAGGGCGATTTAGT | 23S rRNA | 77 | 60 | Courtney et al. (2004) |
|  | Bb23Sr | GCTTCAGCCTGGCCATAAATAG |  |  |  |  |
|  | Bb23Sp | 6-FAM-AGATGTGGTAGACCCGAAGCCGAGTG-TAMRA |  |  |  |  |
|  | IGSa | CGA CCT TCT TCG CCT TAA AGC | rrfA-rrlB intergenic spacer (ITS) | 225–255 | 56 | Derdáková et al. (2003) |
|  | IGSb | AGC TCT TAT TCG CTG ATG GTA-3 |  |  |  |  |
| PCR |  |  |  |  |  |  |
| *Bartonella* spp. | BA325s | CTT CAG ATG ATG ATC CCA AGC CTT CTG GCG | 16S–23S rRNA gene ITS region | 420–780 | 66 | Maggi et al. (2009) |
|  | BA1100as | GAA CCG ACG ACC CCC TGC TTG CAA AGC A | 16S–23S rRNA gene ITS region |  |  |  |
| PCR |  |  |  |  |  |  |
| *Babesia* spp. | BJ1 | GTC TTG TAA TTG GAA TGA TGG | 18S rRNA | 450 | 55 | Casati et al. (2006) |
|  | BN2 | TAG TTT ATG GTT AGG ACT ACG | 18S rRNA |  |  |  |

**Table 3** Correspondence between the obtained *Rickettsia* sequences from *Secretargas transgariepinus* from Jordan and the published sequences acquired from the GenBank

| 17-kDa gene | *Rickettsia* sp. 3 | *Rickettsia* sp. 5 | *Rickettsia* sp. 8 | *Rickettsia* sp. 10 |
| --- | --- | --- | --- | --- |
| *Rickettsia slovaca* isolate Xinjiang-EM (MF002537) | 353/354 bp, 99.72% | 354/354 bp, 100% | 354/354 bp, 100% | 354/354 bp, 100% |
| uncultured *Rickettsia* sp. clone Rs03/FG (KY576907), | 353/354 bp, 99.72% | 353/354 bp, 99.72% | 354/354 bp, 100% | 354/354 bp, 100% |
| *Rickettsia parkeri* isolate RPDPMEX (MG578509), | 352/354 bp, 99.44% | 354/354 bp, 100% | 353/354 bp, 99.72% | 353/354 bp, 99.72% |
| *Rickettsia sibirica* clone XJ-Rick-17kDa-01 (MF098401) | 352/354 bp, 99.44% | 354/354 bp, 100% | 353/354 bp, 99.72% | 353/354 bp, 99.72% |
| *Rickettsia conorii* isolate Xinjiang-PS (MF002513) | 352/354 bp, 99.44% | 354/354 bp, 100% | 353/354 bp, 99.72% | 353/354 bp, 99.72% |
| *Rickettsia africae* isolate Av25 (MH383144) | 352/354 bp, 99.44% | 354/354 bp, 100% | 354/354 bp, 100% | 354/354 bp, 100% |
| *gltA* gene |  |  |  |  |
| *Rickettsia slovaca (AY129301)* |  | 357/358 bp, 99.72% | 358/358 bp, 100% | 358/358 bp, 100% |
| Uncultured *Rickettsia* sp. isolate QH-161 (MG598409) |  | 357/358 bp, 99.72% | 358/358 bp, 100% | 358/358 bp, 100% |
| *Rickettsia sibirica* isolate YC-58 (OM475658) |  | 358/358 bp, 100% | 357/358 bp, 99.72% | 357/358 bp, 99.72 bp |
| *Rickettsia parkeri* isolate RpARAOSA003 (MK814825) |  | 355/355 bp, 100% | 357/358 bp, 99.72% | 357/358 bp, 99.72 bp |
| *Rickettsia conorii* isolate M48 (MF511245) |  | 357/358 bp, 99.72% | 356/358 bp, 99.44% | 356/358 bp, 99.44% |
| *Rickettsia africae* N385 (LC565701) |  | 358/358 bp, 100% | 357/358 bp, 99.72% | 357/358 bp, 99.72% |
| *ompA* gene |  |  |  |  |
| Uncultured *Rickettsia* sp. isolate QH-161 (MG598413) |  | 353/355 bp, 99.44% | 355/356 bp, 99.72% | 356/356 bp, 100% |
| *Rickettsia slovaca* isolate Xinjiang-EM (MF002535) |  | 353/355 bp, 99.44% | 355/356 bp, 99.72% | 356/356 bp, 100% |
| *Rickettsia parkeri* strain LA026 (MH247927) |  | 350/355 bp, 98.59% | 350/355 bp, 98.59% | 351/355 bp, 98.87% |
| *Rickettsia africae* isolate KZN26 (MH751466) |  | 350/355 bp, 98.59% | 351/356 bp, 98.60% | 352/356 bp, 98.88% |
